# Situational Strength Cues from Social Sources at Work: Relative Importance and Mediated Effects

**DOI:** 10.3389/fpsyg.2017.01512

**Published:** 2017-09-05

**Authors:** Balca Alaybek, Reeshad S. Dalal, Zitong Sheng, Alexander G. Morris, Alan J. Tomassetti, Samantha J. Holland

**Affiliations:** ^1^Department of Psychology, George Mason University Fairfax, VA, United States; ^2^C^*2*^ Technologies, Inc. Vienna, VA, United States; ^3^DCI Consulting Group, Inc. Washington, DC, United States

**Keywords:** situational strength, clarity, constraints, coworkers, the immediate supervisor, top management, psychological distance, field theory

## Abstract

Situational strength is considered one of the most important situational forces at work because it can attenuate the personality–performance relationship. Although organizational scholars have studied the consequences of situational strength, they have paid little attention to its antecedents. To address this gap, the current study focused on situational strength cues from different social sources as antecedents of overall situational strength at work. Specifically, we examined how employees combine situational strength cues emanating from three social sources (i.e., coworkers, the immediate supervisor, and top management). Based on field theory, we hypothesized that the effect of situational strength from coworkers and immediate supervisors (i.e., proximal sources of situational strength) on employees' perceptions of overall situational strength on the job would be greater than the effect of situational strength from the top management (i.e., the distal source of situational strength). We also hypothesized that the effect of situational strength from the distal source would be mediated by the effects of situational strength from the proximal sources. Data from 363 full-time employees were collected at two time points with a cross-lagged panel design. The former hypothesis was supported for one of the two situational strength facets studied. The latter hypothesis was fully supported.

## Introduction

“To explain social behavior it is necessary to represent the structure of the total situation and the distribution of the forces in it.”

—Kurt Lewin (1939; p. 868).

As Lewin (1939) stated, a central predictor of human behavior is the situation within which the behavior is enacted. An important characteristic of the situation is its “strength.” Situational strength is defined as “implicit or explicit cues provided by external entities regarding the desirability of potential behaviors” (Meyer et al., 2010). Strong situations can pressure individuals to act in similar ways by providing very clear indicators as to what behavior is most appropriate (Mischel, 1968; Meyer et al., 2010). For example, a red traffic light represents a strong situation in which the appropriate behavior is to stop one's vehicle; in contrast, a yellow traffic light is a weak situation in which some drivers may stop whereas others may attempt to speed through the intersection before the light turns red (Mischel, 1977; Cooper and Withey, 2009). Examples of strong situations in organizational settings might include a formal dress code, an organizational motto such as “The Customer is King/Queen,” and very specific instructions from a supervisor regarding how to perform a task.

Because of its potential to influence (i.e., inhibit or produce) behavioral variation, social scientists have referred to situational strength as “the most important situational moderating variable” (Snyder and Ickes, 1985; p. 904). Within the context of the workplace, situational strength has been conceptualized as a multifaceted construct that includes the clarity of the situational cues from the environment, the consistency of the different situational strength cues, the constraints on the employee's freedom of decision and action, and the consequences of workplace decisions and actions (Meyer et al., 2010, 2014). Meyer et al. (2014) found weak-to-moderate relationships (*r* = −0.22 to 0.49) between the facets and several job characteristics, including feedback (i.e., information from external sources regarding one's performance; Kluger and DeNisi, 1996), role conflict (i.e., the incompatibility or incongruence of different job requirements; Rizzo et al., 1970), autonomy (i.e., “the degree to which the job provides substantial freedom, independence, and discretion to the individual in scheduling the work and determining the procedures to carry it out”; Hackman and Oldham, 1974; p. 258), and production responsibility (i.e., “the cost of errors in terms of both lost output and damage to expensive equipment”; Jackson et al., 1993; p. 754).

These at-best moderate empirical relationships can be explained by the fact that situational strength differs from the other situational constructs in terms of breadth. For example, notwithstanding the similarity in nomenclature, the situational strength facet of constraints is more general than older conceptualizations of constraints (cf. Peters and O'Connor, 1980): although both conceptualizations pertain to a reduction in the number of options available to the employee (due to restrictions imposed by, for example, the supervisor), the older conceptualization further assumes that only *good* options are abridged whereas the situational strength conceptualization is concerned with the extent to which options of *all* kinds are abridged (Meyer et al., 2010). As another example, the situational strength facet of clarity encompasses role clarity (i.e., information that defines the boundaries of the employee's work roles), work behavior prescribed by organizational and societal culture (Gelfand et al., 2006), instructions from the supervisor regarding how to perform tasks properly, and coworker-generated norms regarding backing-up behavior (Meyer et al., 2010, 2014). As these examples suggest, situational strength represents a broad, psychologically-based conceptualization of situational forces applicable across a variety of situational units (e.g., jobs/occupations, roles, tasks, events; Dalal et al., 2014).

Many organizational scientists have highlighted situational strength as an important psychological construct in the workplace because of its outcomes (Johns, 2006; Meyer et al., 2010): most notably, the fact that it weakens the extent to which employee behavior can be predicted via employee personality (Murphy, 2005; Meyer et al., 2014). In particular, several studies have examined situational strength vis-à-vis the validity of the Big Five personality traits (i.e., agreeableness, conscientiousness, extraversion, neuroticism, and openness to experience; McCrae and Costa, 1985; Costa and McCrae, 1992,^1^) in the prediction of job performance. For example, a meta-analysis found that the relationship between the personality trait of conscientiousness and job performance was weaker in occupations characterized by strong situations (e.g., “nuclear equipment operation technicians”; Meyer et al., 2009; p. 1,088) than in occupations characterized by weak situations (e.g., “poets, lyricists, and creative writers”; Meyer et al., 2009; p. 1,088). A more recent meta-analysis found that all the Big Five personality traits were less predictive of job performance in occupations where work processes involved strong situations (e.g., structured work, decision-making constraints) than in occupations where work processes involved weak situations (e.g., unstructured work, decision-making autonomy; Judge and Zapata, 2015). As another example, a recent large-sample study of 17 manufacturing organizations found that the relationship between conscientiousness and employee safety-related behavior was weaker in organizations characterized by strong safety climates (i.e., strong safety-related situations) than in occupations characterized by weak safety climates (i.e., weak safety-related situations; Lee and Dalal, 2016). As for employees' perceptions of overall situational strength on their jobs, a recent field study found that the effects of two personality traits (conscientiousness and agreeableness) on organizational citizenship behavior (i.e., a dimension of job performance defined as voluntary behavior that improves the functioning of the organization and benefits its members; Dalal, 2005) were weaker for employees who perceived the situational strength associated with their job to be high than for those who perceived it to be low (Meyer et al., 2014). Several additional examples exist in the organizational literature (e.g., Barrick and Mount, 1993; Bowling et al., 2015).

Although organizational scientists have acknowledged the importance of the *outcomes* of situational strength on the job (as indicated by the examples in the previous paragraph), little attention has thus far been paid to the *antecedents* of situational strength. To date, three conceptual papers have discussed the potential antecedents of situational strength at work. The first conceptual paper proposed that the strength of societal culture (i.e., the degree to which deviance from norms is tolerated in societies characterized by different cultures) would exert cross-level effects on the strength of organizational cultures (Gelfand et al., 2006). The second conceptual paper proposed that situational strength on the job would be created by (among others) various interpersonal sources in the organization, and would be communicated through channels such as formal policies and procedures and informal norms (Meyer et al., 2010). The third conceptual paper proposed that an individual's perceptions of situational strength cues would be influenced by the strength of the individual's personality (indicated by the consistency of personality-relevant behavior across situations; Dalal et al., 2015). The mechanisms proposed in these conceptual papers have not yet been empirically studied in relation to employees' perceptions of situational strength at work. This is unfortunate because prediction, explanation, and control—the goals of science—require research on antecedents of the construct. Thus, the current study focuses on where (within an organization) the situational strength cues emanate (i.e., sources of situational strength) and how employees combine the situational strength cues emanating from different sources.

Knowledge of the sources of situational strength would provide us with a better psychological understanding of how people experience the situational forces acting on them. This understanding, in turn, would advance situational strength theory. It would also facilitate the more applied goal of shaping situational strength to achieve desired effects. Consider an organization that wishes to create strong situations encouraging the enactment of conscientious *behavior* even by employees who score low on *dispositional* (i.e., trait) conscientiousness (Meyer et al., 2009). For instance, to increase situational strength in terms of social inclusion of individuals with disabilities, it would be beneficial for top management to identify and train allies who can demonstrate inclusive behavior, reemphasize organizational policies, and confront social exclusion at different units in the organization (Sabat et al., 2014). To accomplish this objective successfully, top management would need to understand the situational strength implications of the various human resources practices at different organizational units (Dalal and Meyer, 2012), which is possible by understanding how employees combine the situational strength emanating from different sources.

### Sources of situational strength at work

A number of important sources can influence an employee's perceptions of overall situational strength on the job. Drawing from the literature on reference group and role theories (Gouldner, 1957, 1958; Blau and Scott, 1962; Adams, 1976; Aldrich and Herker, 1977; Salancik and Pfeffer, 1978; Reichers, 1985; Becker, 1992, 2009; Becker and Billings, 1993; Judge and Locke, 1993; Becker et al., 1996), sources of situational strength could be categorized into two broad categories: Internal sources (i.e., sources that are inside the organization, such as coworkers) and external sources (i.e., sources that are outside the organization, such as customers).

The current paper examines the situational strength emanating from three internal sources—namely, coworkers, the immediate supervisor, and top management. These are the three social situational sources that have been most studied by organizational researchers interested in examining employee reactions (e.g., job satisfaction) to various aspects of the work situation (Dalal et al., 2011). The reason extant research on job satisfaction considers these three sources to be particularly worthy of research focus—and the reason we do as well—is that only certain types of employees deal with customers, vendors, or other external social sources, whereas virtually all employees deal with top management, the immediate supervisor, and coworkers (Smith et al., 1969; Dalal et al., 2011). Therefore, research on the three social sources of situational strength should be applicable to virtually all employees in virtually all organizations.

We use field theory (Lewin, 1939, 1943, 1951) to examine how employees make sense of the behavioral cues emanating concurrently from these three social sources to form a unified perception of situational strength on the job. Field theory posits that the environment surrounding an individual can be conceived of as a field or system of forces, and that the behavior of an individual in a specific situation is a function of the individual's personality and various situational forces (Lewin, 1939, 1943). Situations may emerge as barriers and opportunities to express behavior (Lewin, 1951); these represent strong and weak situations, respectively (Mischel, 1968). Here, we elaborate on how situational strength from different sources might emerge in an organizational setting.

Organizational research has long established that organizations are social systems (Katz and Kahn, 1978) consisting of interrelated entities such as employees, clients, and managers (Blau and Scott, 1962; Salancik and Pfeffer, 1978). Organizational entities (also called “role senders”) seek to motivate employees to behave in certain ways to achieve work-related goals (Reichers, 1985). As such, organizational entities compose various sources of situational strength, varying in their nature and in the level of abstraction of informational cues guiding employee behavior.

The three social sources, which are the focus of the current study, can be distinguished on the basis of theories of organizational structure (e.g., Stratified Systems Theory; Jaques, 1976). These theories posit that organizations can be viewed as having five hierarchical levels: Front-line employees, first-line supervisors, middle managers, directors, and top management (or “C-suite” executives such as the Chief Executive Officer and the Chief Financial Officer). The focal employees in the current paper are employees who fall within any of the first three hierarchical levels. Moreover, based on previous research showing that employees do not make fine-grained distinctions between specific levels of management higher than their immediate supervisor (Herzberg et al., 1957; Dalal et al., 2011), the current paper distinguishes only between the immediate supervisor and top management.

Coworkers act as the employee's social and task partners (Chiaburu and Harrison, 2008). Immediate supervisors allocate tasks, provide performance feedback, and communicate the organizational goals set by top management (Jacobs and McGee, 2001; Zaccaro and Klimoski, 2001). Top management creates and communicates strategic vision throughout the organization (Jacobs and McGee, 2001; Zaccaro and Klimoski, 2001) and influences the behavior of employees through interventions geared toward formal reward systems, technological factors (e.g., work flow process), physical settings (e.g., architectural design), and social factors (e.g., interactive processes at the individual, group, and intergroup levels; Cardy and Selvarajan, 2001). As a result of these differences in the functions and communications of the sources of situational strength, each source should provide a *part* of the *whole* amount of situational strength exerted on an employee (Locke, 1976; Judge and Locke, 1993).

Indirect evidence for this notion comes from two meta-analyses. The first meta-analysis demonstrated that contextual variables from various sources (e.g., the leader, task properties) typically exhibit incremental validity over each other vis-à-vis criteria such as job attitudes and performance (Podsakoff et al., 1996). The second meta-analysis demonstrated that social influences from coworkers provide incremental validity beyond social influences from leaders vis-à-vis criteria such as job involvement and withdrawal (Chiaburu and Harrison, 2008). As such, we predict that all three sources of situational strength contribute uniquely to employee perceptions of overall situational strength:

***Hypothesis 1:*** *Perceptions of situational strength from coworkers, the immediate supervisor, and top management explain unique variance in perceptions of overall situational strength on the job*.

Moreover, according to field theory, the psychological distances of environmental factors from the employee predict their level of impact on the employee's behavior (Lewin, 1943). Specifically, psychologically proximal factors tend to exert stronger effects than psychologically distal factors. In organizations, psychological distance has been conceptualized as the frequency of meaningful interaction: the greater the frequency of meaningful interactions an employee has with a source, the more proximal the source should become to the employee (Becker, 2009). Employees likely work in closer physical proximity to and have more frequent interactions with coworkers and immediate supervisors than with top management (Allen, 1977; Sias and Cahill, 1998). As a result of close proximity and shared goals, employees develop close friendships with their coworkers (Sias and Cahill, 1998), and they report stronger feelings of attachment and identification with both coworkers and immediate supervisors than with top management (Becker, 1992). Moreover, top management can be distanced from the employees in terms of hierarchical rank or social status, suggesting that employees have a lower sense of attachment and identification with top management (Bloom, 1999; Halevy et al., 2011, 2012).

These findings suggest that employees have more frequent meaningful interactions with their coworkers and immediate supervisors than with their top management. In sum, coworkers and the immediate supervisor are considered to be more proximal sources of situational strength and top management is considered to be a more distal source of situational strength. We therefore suggest that psychologically proximal sources of situational strength have stronger effects than psychologically distal sources on employees' perceptions of overall situational strength on the job:

***Hypothesis 2:*** *Psychological proximity positively predicts perceptions of overall situational strength on the job, such that the effect of more proximal sources (i.e., situational strength attributable to coworkers and the immediate supervisor) on overall situational strength is stronger than the effect of the more distal source (i.e., situational strength attributable to top management)*.

Another characteristic of the work situation in general, and therefore situational strength in particular, is that the situation at one level of analysis can influence the situation at another level, and that this multilevel influence can manifest in a top-down manner (Johns, 2006). Along these lines, the distal-proximal framework of motivational theories posits that the influence of distal motivational predictors is transmitted *through* proximal motivational predictors (Kanfer, 1991). Extant applications of this approach relevant to the current study are found in organizational leadership research. For example, Osborn et al. (2002) postulated that the stronger a top leader's connections with his or her subordinate managers, the more likely he or she would be to influence the work environment of those at the bottom of the organizational hierarchy. Berson and Avolio (2004) showed that information regarding strategic organizational goals (which are set by CEOs) was disseminated to department managers through vice presidents and division managers. Similarly, Mayer et al. (2009) showed that the ethical leadership behavior of supervisors mediates the relationships between the ethical leadership behavior of top management and outcomes (i.e., organizational citizenship behavior and counterproductive work behavior) at lower levels of the organizational hierarchy. As such, we hypothesize the following:

***Hypothesis 3:*** *The relationship between perceptions of situational strength (SS) from the distal source (i.e., top management) and perceptions of overall situational strength is mediated by perceptions of situational strength from proximal sources (i.e., coworkers and immediate supervisor)*.

**Figure 1:**
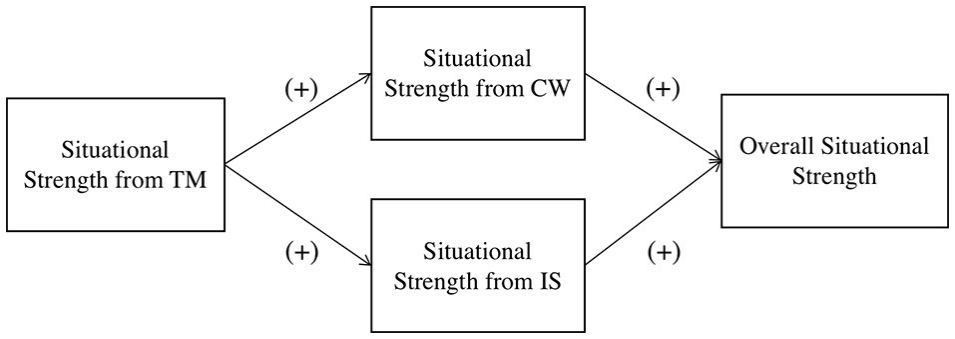
Conceptual model. CW, Coworkers; IS, The immediate supervisor; TM, Top management.

The overall conceptual model is displayed in Figure 1.

## Methods

### Participants and procedure

We recruited respondents through Amazon.com's Mechanical Turk (MTurk), an online labor market where requesters (e.g., researchers) recruit workers (e.g., respondents) for the completion of human intelligence tasks (e.g., surveys) in exchange for compensation. Research has shown that MTurk participants provide data at least as reliable as data obtained through traditional research methods (Buhrmester et al., 2011; Holden et al., 2013). In recent years, MTurk has become an increasingly popular recruitment tool among social scientists (Amir et al., 2012; Fast et al., 2012; Crump et al., 2013; Giacopelli et al., 2013) because it provides instant access to a respondent pool with wide demographic diversity (Ipeirotis, 2010; Casler et al., 2013).

We used a cross-lagged panel design (Farrell, 1994; Kline, 2011) to collect survey data on the focal variables at two time points, separated by 2 weeks^2^. In accordance with this design, the focal variables (i.e., overall situational strength and situational strength from the three sources) were measured at both time points (see Table 1 for details), and all the other variables (i.e., demographics, frequency of interaction with the sources, and identification with the sources) were measured at Time 1 only. Sample sizes for Time 1 and Time 2 were 451 and 372, respectively (82% retention rate across time points). Nine cases were excluded because they showed one or more abnormal response patterns (Johnson, 2005; Huang et al., 2012) associated with insufficient effort responding: namely, had more than 50% missing data, included the same response to all situational strength items, and/or had survey completion times greater than two standard deviations from the mean (McGrath et al., 2010). Elimination of these 9 cases led to an effective sample size of 363, which included four cases with less than 50% missing data. Individual analyses were conducted with complete cases (using listwise deletion of missing data). The sample sizes for individual analyses after listwise deletion varied between 359 and 363^3^.

**Table 1:** Situational strength measure modified from Meyer et al. (2014).

	Situational strength from sources^*^	Overall situational strength^**^
Clarity items^***^	…provided you with specific information about your work-related responsibilities? …provided you with straightforward information about what you need to do to succeed? …provided you with easy-to-understand information about work requirements? …told you exactly what to expect at work (on your job)? …provided you with information about how to properly do your job? …told you exactly what is expected from you at work (on your job)? …provided you specific information about which tasks to complete?	…have you been provided with specific information about your work-related responsibilities? …have you been provided with straightforward information about what you need to do to succeed? …have you been provided with easy-to-understand information about work requirements? …have you been told exactly what to expect on your job? …have you been provided with information about how to properly do your job? …have you been told exactly what is expected from you on your job? …have you been provided with specific information about which tasks to complete?
Constraints items^***^	…prevented you from making your own decisions? …applied constraints that prevented you from doing things in your own way? …prevented you from choosing how to do things? …limited your freedom to make decisions? …applied procedures that prevented you from working your own way? …limited what you could do? …restricted when or how you could do things?	…have you been prevented from making your own decisions? …have constraints been applied that prevented you from doing things in your own way? …have you been prevented from choosing how to do things? …your freedom to make decisions has been limited? …have procedures been applied that prevented you from working your own way? …has what you could do been limited? …has when or how you could do things been restricted?

* Time 1 prompt, To what extent have (has) coworkers with whom you interact most frequently/your immediate supervisor/top management in your organization.…Time 2 prompt, During the past week, to what extent have (has) coworkers with whom you interact most frequently/your immediate supervisor/top management in your organization…

** Time 1 prompt, Think about your job as a whole. Overall (all things considered), to what extent…Time 2 prompt, Think about your job as a whole during the past week. Overall (all things considered), to what extent…

*** *Response Scale. 1, Not at all; 2, To a slight extent; 3, To a moderate extent; 4, To a large extent; 5, To a very large extent*.

The final sample consisted of 55% U.S. and 45% Indian employees, was 29% female, and had a mean age of 33 years (SD = 9.30 years). As per the requirements for participation in the study, all respondents were fluent in English, worked at least 30 h per week in an organization with at least 50 employees (i.e., had a sufficient number of coworkers), and had at least two levels of management above them (i.e., had separate immediate supervisor and top management personnel).

The survey was administered using Qualtrics (http://www.qualtrics.com), an online platform for researchers to develop and administer survey questionnaires through the Internet. For reasons discussed subsequently, two facets of situational strength were assessed: clarity and constraints (Meyer et al., 2010). For each facet of situational strength, we measured perceived situational strength emanating from three sources (coworkers, the immediate supervisor, and top management) as well as overall situational strength. Situational strength items for each facet-source combination were presented on a separate page of the survey. The order of the pages, and of the items within a page, was randomized so as to prevent order effects.

### Measures

We measured perceived situational strength using an adapted version of Meyer et al.'s Situational Strength at Work (SSW) scale (Meyer et al., 2014). The original scale included four facets of situational strength (i.e., clarity, constraints, consistency, and consequences). However, in the current study, due to the need to measure multiple *sources* of situational strength associated with each *facet* of situational strength, survey length constraints precluded the possibility of assessing all four facets of situational strength.

We therefore included measures of one “positive” and one “negative” facet of situational strength (see José et al., 2011). A positive facet of situational strength is one that is related positively to job attitudes (e.g., job satisfaction and organizational commitment) and one for which an inadequate supply of situational strength (i.e., preferred levels < actual levels) leads to worse job attitudes than an excess supply (i.e., actual levels > preferred levels). A negative facet, in contrast, is one that is related negatively to job attitudes and one for which an excess supply of situational strength leads to worse job attitudes than an inadequate supply. The situational strength facet of consequences (i.e., the extent to which workplace decisions and/or actions have important implications; Meyer et al., 2010) can involve both positive and negative consequences to the employee; hence, we did not include it.

We also did not include the facet of consistency. This facet (a positive situational strength facet) is typically defined as the congruency *across* various sources of situational strength (Meyer et al., 2010). As a result, this facet is incompatible with the current paper's purpose of “unpacking” the various sources of situational strength.

We therefore focused on the remaining two facets of situational strength: clarity (i.e., “the extent to which cues regarding work-related responsibilities or requirements are available and easy to understand”, Meyer et al., 2010; p. 125) and constraints (i.e., “the extent to which an individual's freedom of decision and action is limited by forces outside his or her control”, Meyer et al., 2010; p. 126). See Table 1 for items, instructions, and response options.

Psychological distance from the sources of situational strength was assessed via frequency of interaction and identification with sources of situational strength. See Table 2 for items and response options. See Table 3 for descriptive statistics, scale reliabilities, and inter-correlations for all measures.

**Table 2:** Measures of frequency of interaction and identification with sources of situational strength.

	**Item(s)**
Frequency of interaction with the sources^*^	How frequently do you interact with your coworkers/your immediate supervisor/top management in your organization?
Identification with the sources^**^	When someone criticizes [the source of situational strength]^***^, it feels like a personal insult. When I talk about [the source of situational strength], I usually say “we” rather than “they.” The successes of [the source of situational strength] are my successes. When someone praises [the source of situational strength], it feels like a personal compliment. I feel a sense of “ownership” for [the source of situational strength].

*Frequency of interaction and identification with the sources were measured at Time 1 only*.

* *Response Scale. 1, Never; 2, Less than once a month; 3, Once a Month; 4, 2–3 Times a Month; 5, Once a Week; 6, 2–3 Times a Week; 7, Daily*.

** *Items were adapted from Becker's (1992) organizational identification measure. Response Scale: 0, Not Applicable; 1, Strongly Disagree; 2, Somewhat Disagree; 3, Slightly Disagree; 4, Neither Agree nor Disagree; 5, Slightly Agree; 6, Somewhat Agree; 7, Strongly Agree*.

*** *The source of situational strength = The coworkers with whom I interact most frequently/my immediate supervisor/the top managers in my organization*.

**Table 3:** Descriptive statistics, scale reliabilities, and inter-correlations.

**Variables**	**M**	***SD***	**1**	**2**	**3**	**4**	**5**	**6**	**7**	**8**	**9**	**10**	**11**	**12**	**13**	**14**	**15**	**16**	**17**	**18**	**19**	**20**	**21**	**22**
1. Frequency of interaction with CW	6.71	0.78	–																					
2. Frequency of interaction with IS	6.40	1.01	0.62^**^	–																				
3. Frequency of interaction with TM	4.28	1.65	0.09	0.19^**^	–																			
4. Identification with CW	5.00	1.33	0.03	0.13^*^	0.08	(0.84)																		
5. Identification with IS	4.27	1.63	−0.07	0.01	0.20^**^	0.58^**^	(0.91)																	
6. Identification with TM	3.72	1.85	−0.18^**^	−0.06	0.31^**^	0.49^**^	0.77^**^	(0.94)																
7. Clarity from CW T1	3.20	0.96	−0.01	0.01	0.06	0.33^**^	0.32^**^	0.34^**^	(0.94)															
8. Clarity from IS T1	3.70	0.81	0.14^**^	0.20^**^	0.03	0.16^**^	0.24^**^	0.10	0.33^**^	(0.93)														
9. Clarity from TM T1	3.09	1.07	−0.02	0.03	0.35^**^	0.32^**^	0.39^**^	0.50^**^	0.35^**^	0.40^**^	(0.96)													
10. Overall Clarity T1	3.60	0.84	0.12^*^	0.20^**^	0.09	0.29^**^	0.20^**^	0.14^**^	0.42^**^	0.68^**^	0.47^**^	(0.93)												
11. Constraints from CW T1	1.87	0.92	−0.26^**^	−0.29^**^	0.15^**^	0.05	0.24^**^	0.33^**^	0.14^**^	−0.16^**^	0.14^**^	−0.19^**^	(0.96)											
12. Constraints from IS T1	2.48	0.92	−0.09	−0.12^*^	0.08	−0.04	−0.11^*^	0.04	0.04	−0.18^**^	0.02	−0.14^**^	0.59^**^	(0.95)										
13. Constraints from TM T1	2.59	1.02	−0.09	−0.11^*^	0.00	0.04	−0.09	−0.07	0.05	−0.16^**^	0.00	−0.16^**^	0.47^**^	.067^**^	(0.96)									
14. Overall Constraints T1	2.60	0.90	−0.08	−0.11^*^	−0.02	−0.02	−0.13^*^	−0.09	0.02	−0.16^**^	−0.03	−0.14^**^	0.53^**^	0.75^**^	0.76^**^	(0.95)								
15. Clarity from CW T2	2.75	1.09	−0.07	−0.07	0.25^**^	0.30^**^	0.37^**^	0.45^**^	0.43^**^	0.09	0.43^**^	0.14^**^	0.25^**^	0.12^*^	0.18^**^	0.12^*^	(0.96)							
16. Clarity from IS T2	3.18	0.95	0.05	0.07	0.16^**^	0.22^**^	0.36^**^	0.35^**^	0.32^**^	0.46^**^	0.43^**^	0.40^**^	0.01	−0.04	−0.01	−0.05	0.53^**^	(0.94)						
17. Clarity from TM T2	2.64	1.17	−0.07	−0.08	0.34^**^	0.26^**^	0.44^**^	0.57^**^	0.34^**^	0.16^**^	0.59^**^	0.20^**^	0.27^**^	0.08	0.08	0.01	0.66^**^	0.61^**^	(0.96)					
18. Overall Clarity T2	3.08	0.96	−0.05	−0.02	0.22^**^	0.29^**^	0.40^**^	0.43^**^	0.40^**^	0.35^**^	0.50^**^	0.42^**^	0.10	−0.02	0.01	−0.03	0.62^**^	0.78^**^	0.65^**^	(0.94)				
19. Constraints from CW T2	1.77	0.96	−0.22^**^	−0.24^**^	0.23^**^	0.02	0.23^**^	0.30^**^	0.13^*^	−0.20^**^	0.15^**^	−0.14^**^	0.63^**^	0.42^**^	0.36^**^	0.41^**^	0.33^**^	0.12^*^	0.38^**^	0.19^**^	(0.97)			
20. Constraints from IS T2	2.22	1.02	−0.15^**^	−0.14^**^	0.13^*^	0.00	0.00	0.10	0.09	−0.16^**^	0.08	−0.16^**^	0.53^**^	0.61^**^	0.54^**^	0.59^**^	0.27^**^	0.07	0.23^**^	0.13^*^	0.69^**^	(0.96)		
21. Constraints from TM T2	2.15	1.01	−0.15^**^	−0.15^**^	0.08	0.05	0.06	0.13^*^	0.14^**^	−0.17^**^	0.08	−0.19^**^	0.51^**^	0.48^**^	0.58^**^	0.55^**^	0.26^**^	0.10	0.27^**^	0.13^*^	0.64^**^	0.76^**^	(0.96)	
22. Overall Constraints T2	2.30	0.98	−0.16^**^	−0.15^**^	0.05	−0.02	0.03	0.10^*^	0.12^*^	−0.19^**^	0.06	−0.16^**^	0.55^**^	0.56^**^	0.49^**^	0.59^**^	0.28^**^	0.11^*^	0.26^**^	0.16^**^	0.68^**^	0.82^**^	0.77^**^	(0.96)

*M, Mean; SD, Standard deviation; CW, Coworkers; IS, The immediate supervisor; TM, Top management; T1, Time 1; and T2, Time 2; Overall Clarity/Constraints, Perceptions of overall clarity/constraints on the job*.

*N = 362–363*.

* *p < 0.05*,

** *p < 0.01*.

We additionally assessed longitudinal measurement invariance (in Mplus 7) for the situational strength measures so as to ensure that respondents interpreted these measures in a conceptually similar manner at both time points. According to the approach recommended by Vandenberg and Lance (2000), we tested for longitudinal measurement invariance with a confirmatory factor analysis (CFA) approach. First, to establish a baseline fit (also referred to as configural invariance), we conducted a multi-sample analysis with the same factor structure within each group but no invariance restriction on loadings. Next, to evaluate whether the participants attributed the same meanings to the latent constructs across time points (also referred to as metric invariance), we re-estimated the measurement model with an equality constraint placed upon factor loadings across two time points. To evaluate whether the meanings and the mean levels of the latent constructs remained the same across time points, we tested the scalar invariance by setting factor loadings as well as intercepts to be equal across time points^4^. As can be seen in Table 4, metric invariance was demonstrated for both clarity and constraints (with scalar invariance furthermore being demonstrated for constraints), indicating that the latent constructs of situational strength (i.e., clarity and constraints) were equally well represented across both time points by the measure used in the study.

**Table 4:** Longitudinal measurement invariance results.

	**Test**	**χ^2^**	**df**	**CFI**	**TLI**	**RMSEA**	**SRMR**	***Δχ*^2^**
Clarity	Baseline^a^	2983.875	1,456	0.924	0.920	0.054	0.038	
	Metric Invariance^b^	3002.157	1,480	0.924	0.921	0.054	0.039	18.282 (*p* > 0.05)
	Scalar Invariance^c^	3045.119	1,504	0.923	0.922	0.053	0.040	42.962 (*p* < 0.05)
Constraints	Baseline^a^	3178.261	1,456	0.927	0.923	0.057	0.029	
	Metric Invariance^b^	3201.797	1,480	0.927	0.924	0.057	0.031	23.536 (*p* > 0.05)
	Scalar Invariance^c^	3235.698	1,504	0.927	0.925	0.057	0.031	33.901 (*p* > 0.05)
	Strict Invariance^d^	3313.727	1,532	0.924	0.924	0.057	0.032	78.029 (*p* < 0.05)

*N = 359. Red colored font indicates the highest level of invariance achieved*.

*χ^2^, Chi square statistic; df, Degrees of freedom; CFI, Comparative Fit Index; TLI, Tucker-Lewis Index; RMSEA, Root Mean Square Error of Approximation; SRMR, Root Mean Square Residual*.

a *Baseline fit was established via a multi–sample analysis with no longitudinal invariance restriction*.

b *Metric invariance was tested by placing equality constraints upon factor loadings across the two time points. Support for metric invariance indicated that participants attributed the same meanings to the latent constructs across time points*.

c *Scalar invariance was tested by setting factor loadings and intercepts to be equal across the two time points. Support for scalar invariance indicated that the meanings and the mean levels of the latent constructs remained the same across time points*.

d *Strict invariance was tested by adding an additional constraint of equal errors across the two time points*.

## Results

***Hypothesis 1*** stated that perceptions of situational strength from each source would explain unique variance in perceptions of overall situational strength on the job. ***Hypothesis 2*** built on this by stating that the effects of proximal sources of overall situational strength on the job would be stronger than those of the distal source. The assumption underlying Hypothesis 1 was that employees would distinguish among situational strength cues emanating from different sources. We began by testing this assumption with CFA. The results (see Table 5) showed that, for both clarity and constraints at both time points, a 4-factor model with situational strength from coworkers, situational strength from the immediate supervisor, situational strength from top management, and overall situational strength as distinct factors fit the data well and significantly better than not only a 1-factor model (whereby all situational strength items of a facet at a given time load on a single construct) but also all possible 3-factor models (all chi-squared difference tests yielded *p* < 0.01). The assumption that employees would distinguish among situational strength from different sources was therefore supported.

**Table 5:** Confirmatory Factor Analysis (CFA) results for Hypothesis 1.

	**Model**	**χ^2^**	**df**	**CFI**	**TLI**	**RMSEA**	**SRMR**	***Δχ*^2^**
Clarity time 1	4-factor	822.665	344	0.935	0.928	0.067	0.038	
	3-factor Alt1	2203.662	347	0.747	0.724	0.131	0.165	1380.997^**^
	3-factor Alt2	2072.809	347	0.764	0.743	0.126	0.139	1250.144^**^
	3-factor Alt3	1351.327	347	0.863	0.851	0.096	0.058	528.662^**^
	3-factor Alt4	2276.679	347	0.737	0.713	0.133	0.137	1454.014^**^
	3-factor Alt5	2114.751	347	0.759	0.737	0.120	0.109	1292.086^**^
	3-factor Alt6	1997.366	347	0.775	0.755	0.123	0.131	1174.701^**^
	1-factor	4904.150	350	0.488	0.447	0.190	0.156	4081.485^**^
Constraints time 1	4-factor	798.366	344	0.946	0.941	0.065	0.031	
	3-factor Alt1	2015.223	347	0.801	0.783	0.124	0.112	1216.857^**^
	3-factor Alt2	1730.519	347	0.835	0.820	0.113	0.068	932.153^**^
	3-factor Alt3	1364.388	347	0.879	0.868	0.097	0.054	566.022^**^
	3-factor Alt4	2437.331	347	0.751	0.729	0.139	0.151	1638.965^**^
	3-factor Alt5	2110.446	347	0.791	0.771	0.127	0.142	1312.08^**^
	3-factor Alt6	1347.877	347	0.881	0.870	0.096	0.053	549.511^**^
	1-factor	4045.265	350	0.646	0.617	0.171	0.115	3246.899^**^
Clarity time 2	4-factor	776.715	344	0.948	0.943	0.063	0.035	
	3-factor Alt1	1920.393	347	0.812	0.795	0.120	0.095	1143.678^**^
	3-factor Alt2	1821.326	347	0.824	0.808	0.116	0.098	1044.611^**^
	3-factor Alt3	1033.127	347	0.918	0.911	0.079	0.045	256.412^**^
	3-factor Alt4	1808.275	347	0.825	0.809	0.116	0.071	1031.56^**^
	3-factor Alt5	1718.364	347	0.836	0.821	0.112	0.082	941.649^**^
	3-factor Alt6	1723.755	347	0.835	0.820	0.112	0.089	947.04^**^
	1-factor	3991.200	350	0.652	0.624	0.170	0.105	3214.485^**^
Constraints time 2	4-factor	882.375	344	0.944	0.939	0.071	0.030	
	3-factor Alt1	2027.331	347	0.826	0.811	0.124	0.086	1144.956^**^
	3-factor Alt2	1691.157	347	0.861	0.848	0.111	0.054	808.782^**^
	3-factor Alt3	1332.598	347	0.898	0.889	0.095	0.041	450.223^**^
	3-factor Alt4	2272.478	347	0.801	0.783	0.133	0.090	1390.103^**^
	3-factor Alt5	1941.864	347	0.835	0.820	0.121	0.091	1059.489^**^
	3-factor Alt6	1564.777	347	0.874	0.863	0.106	0.053	682.402^**^
	1-factor	3934.146	350	0.707	0.684	0.169	0.084	3051.771^**^

*N = 359–363*.

** *p < 0.01*.

*χ^2^, Chi square statistic; df, Degrees of freedom; CFI, Comparative Fit Index; TLI, Tucker-Lewis Index; RMSEA, Root Mean Square Error of Approximation; SRMR, Root Mean Square Residual*.

*3-factor Alt1 = IS (the immediate supervisor) and CW (coworkers) combined as one factor; 3-factor Alt2, IS and TM (top management) combined as one factor; 3-factor Alt3, IS and OA (overall) combined as one factor; 3-factor Alt4, CW and TM combined as one factor; 3-factor Alt5, CW and OA combined as one factor; 3-factor Alt6, TM and OA combined as one factor*.

The assumption underlying Hypothesis 2 was that coworkers and the immediate supervisor were more proximal sources of situational strength and that top management was a more distal source of situational strength. Mean levels of frequency of interaction and identification with the sources of situational strength (see Table 3) and comparisons of the means for proximal vs. distal sources (see Table 6), which showed significant differences (*p* < 0.01), revealed support for this assumption.

**Table 6:** Frequency of interaction and identification: comparison of proximal vs. distal sources.

**Comparison**	**Mean difference**	**Standard deviation**	**Standard error mean**	**Confidence interval of difference**	**t (df)**
Frequency of interaction with CW vs. TM	2.44	1.77	0.09	[2.25, 2.62]	26.27^**^ (362)
Frequency of interaction with IS vs. TM	2.12	1.76	0.09	[1.94, 2.30]	22.95^**^ (362)
Identification with CW vs. TM	1.28	1.67	0.09	[1.11, 1.45]	14.61^**^ (362)
Identification with IS vs. TM	0.55	1.20	0.06	[0.42, 0.67]	8.65^**^ (362)

*N = 363. df, Degrees of freedom*.

** *p < 0.01*.

*CW, Coworkers; IS, The immediate supervisor; TM, Top management. Mean Difference > 0 (which was always the case here) means that frequency of interaction and identification are higher for CW or IS than for TM*.

Moreover, as can be seen in Table 3, situational strength scores from the three sources (i.e., the predictors in this study) were significantly (*p* < 0.05) and positively inter-correlated. When using multiple, meaningfully correlated predictors, both bivariate correlations and regression coefficients from the full model (containing all predictors) can provide misleading conclusions regarding the relative importance of predictors—and therefore a technique such as relative weight analysis is preferred (LeBreton et al., 2007). Relative weight analysis reveals the unique contribution of each predictor variable to the overall model R^2^ considering the existence of other predictors (LeBreton et al., 2007). We used Tonidandel and LeBreton (2015) RWA-WEB tool to obtain the relative weight of proximal vs. distal sources of situational strength—and thereby to test Hypotheses 1 and 2.

Analyses were performed separately for the clarity and the constraints facets of situational strength. In all analyses, the criterion (i.e., overall clarity or overall constraints) measured at Time 2 was regressed on the predictors (i.e., clarity or constraints from the sources) measured at Time 1, with overall situational strength measured at Time 1 used as a statistical control, as recommended for evaluating causal relationships in two-wave longitudinal studies (Cole and Maxwell, 2003).

Table 7 summarizes the relative weight analysis results. For comparison purposes, standardized regression weights (β) are also reported. The lower bound of the 95% confidence interval for the relative weights excluded zero for every source of the clarity and constraints facets of situational strength—thereby supporting Hypothesis 1. In contrast, support for Hypothesis 2 depended on the facet of situational strength under consideration. For clarity, the rescaled relative weights for coworkers and the immediate supervisor were actually lower than the rescaled relative weight for top management—thereby falsifying Hypothesis 2. For constraints, however, the rescaled relative weights for coworkers and the immediate supervisor were higher than the rescaled relative weight for top management—thereby supporting Hypothesis 2.

**Table 7:** Summary of relative weight analyses for the effect of situational strength from sources on overall situational strength.

**Predictor**	**β**	**RW**	**CI-L**	**CI-U**	**RS-RW (%)**
**CRITERION** = **OVERALL CLARITY T2 [R**^**2**^ = **0.33;** ***F***_**(4, 358)**_ = **43.45;** ***p*** < **0.001]**					
Clarity from CW T1	0.20	0.08	0.0353	0.1288	23.59
Clarity from IS T1^a^	0.05	0.04	0.0164	0.0744	11.94
Clarity from TM T1	0.34	0.14	0.0854	0.2141	44.47
Overall clarity T1	0.14	0.06	0.0301	0.1118	20.00
**CRITERION** = **OVERALL CONSTRAINTS T2 [R**^**2**^ = **0.43;** ***F***_**(4, 358)**_ = **68.51;** ***p*** < **0.001]**					
Constraints from CW T1^b^	0.29	0.13	0.0855	0.1913	30.78
Constraints from IS T1	0.14	0.10	0.0661	0.1430	23.73
Constraints from TM T1	0.03	0.07	0.0407	0.1065	16.52
Overall constraints T1	0.31	0.13	0.0798	0.1738	28.97

*Overall Clarity/Constraints, Perceptions of overall clarity/constraints on the job; T1, Time 1; T2, Time 2; CW, Coworkers; IS, The immediate supervisor; TM, Top management; β, standardized regression weight; RW, raw relative weights that sum to the R^2^ of the model; CI-L, lower bound of confidence interval for the raw relative weights; CI-U, upper bound of confidence interval for the raw relative weights; RS-RW (%), relative weights rescaled as percentages of the R^2^ of the model, which sum to 100%*.

a *The relative weight for this variable significantly differs (CI = −0.1804 to −0.0392) from the relative weight of Clarity-TM (T1)*.

b *The relative weight for this variable significantly differs (CI = 0.0006 to 0.1314) from the relative weight of Constraints-TM (T1)*.

*The 95% confidence intervals (CIs)—corresponding to a significance test at alpha = 0.05—for the individual weights of situational strength from the sources and all corresponding significance tests were based on bootstrapping with 10,000 iterations, as recommended by Tonidandel et al. (2009)*.

**Figure 2:**
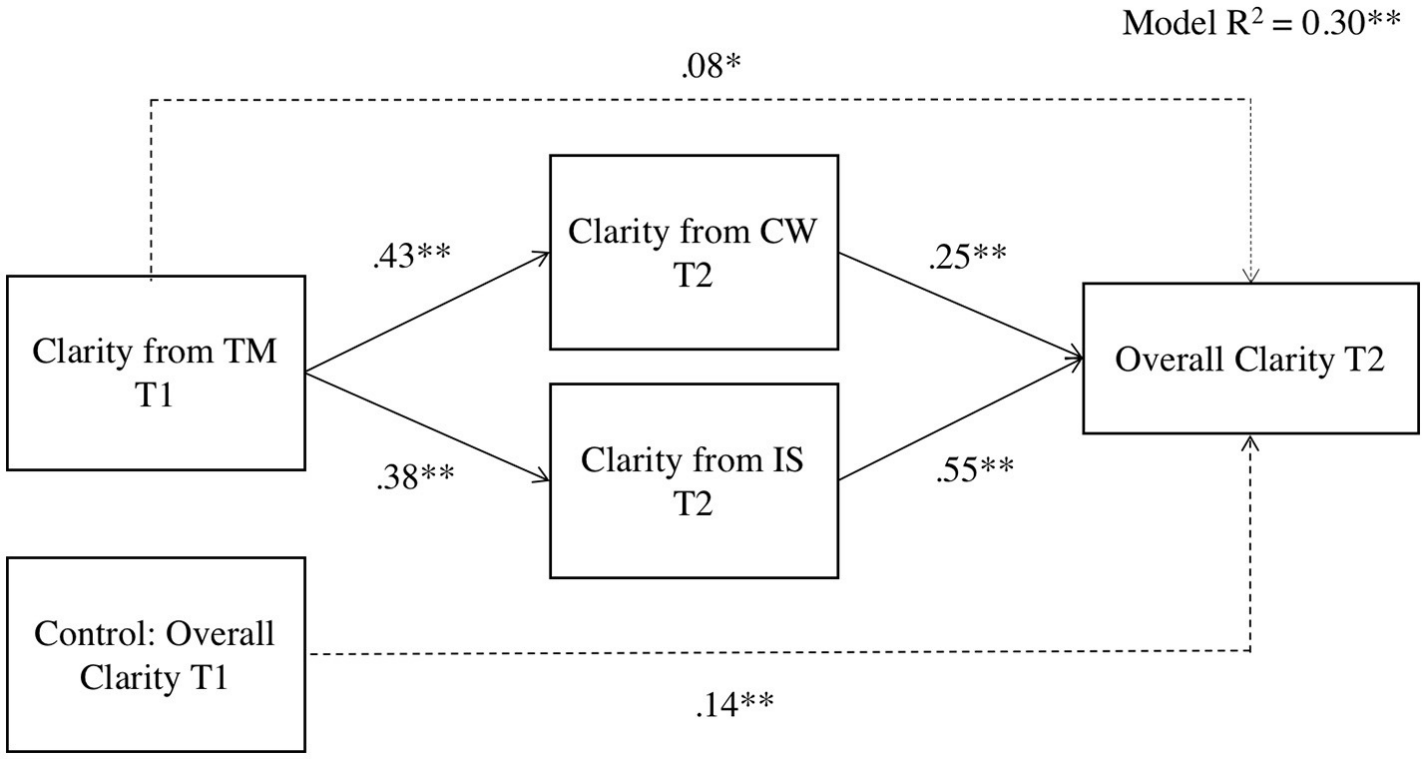
PROCESS results for clarity facet of situational strength. *N* = 363; ^*^p < 0.05, ^**^ *p* < 0.01; CW, Coworkers; IS, The immediate supervisor; TM, Top management; T1, Time 1; T2, Time 2; Overall Clarity, Perceptions of overall clarity on the job.

**Figure 3:**
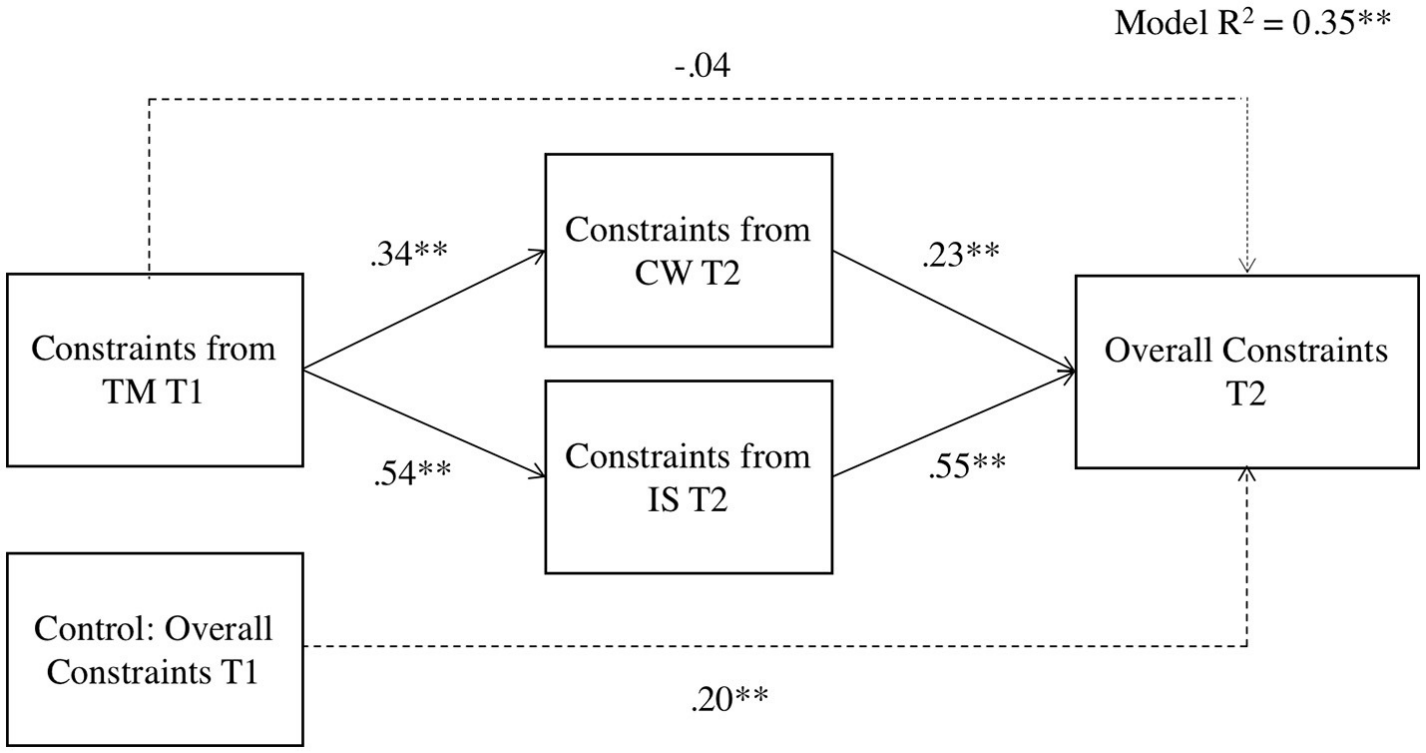
PROCESS results for constraints facet of situational strength. *N* = 362; ^*^p < 0.05, ^**^p < 0.01; CW, Coworkers; IS, The immediate supervisor; TM, Top management; T1, Time 1; T2, Time 2; Overall Constraints, Perceptions of overall constraints on the job.

***Hypothesis 3***, which stated that the relationship between perceptions of situational strength from the distal source and perceptions of overall situational strength would be mediated by the perceptions of situational strength from proximal sources, was tested separately for clarity and constraints via mediation analyses in SPSS using Hayes's PROCESS macro with 1,000 bootstrap samples (Hayes, 2013). Following recommendations for longitudinal mediation (Cole and Maxwell, 2003; MacKinnon et al., 2007), we tested two mediation models (summarized in Figures 2, 3 and Tables 8, 9). Figure 2 and Table 8 show that the effect of perceptions of clarity from top management on perceptions of overall clarity is partially mediated by perceptions of clarity from coworkers and the immediate supervisor. Figure 3 and Table 9 show that the effect of perceptions of constraints from top management on perceptions of overall constraints is fully mediated by perceptions of constraints from coworkers and the immediate supervisor. In sum, Hypothesis 3 is supported^5^.

**Table 8:** Mediation of the effect of clarity from top management on overall clarity through clarity from the immediate supervisor and from coworkers.

	**Point estimate**	**Products of coefficient**		**Percentile 95% CI**	
		**S.E**.	***t***	**Lower**	**Upper**
**DIRECT EFFECT**					
	0.08	0.03	2.36^**^	0.01	0.14
**INDIRECT EFFECTS**					
Clarity from CW	0.11	0.02	5.71^**^	0.07	0.15
Clarity from IS	0.21	0.03	7.51^**^	0.16	0.27
TOTAL	0.31	0.03	9.36^**^	0.25	0.39

*N = 363*.

** *p < 0.01*.

*CI, Confidence interval; S.E., Standard error; CW, Coworkers; IS, The immediate supervisor. Control variable = Perceptions of overall clarity on the job at Time 1*.

**Table 9:** Mediation of the effect of constraints from top management on overall constraints through constraints from the immediate supervisor and from coworkers.

	**Point estimate**	**Products of coefficient**		**Percentile 95% CI**	
		**S.E**.	***T***	**Lower**	**Upper**
**DIRECT EFFECT**					
	−0.04	0.04	−0.84	−0.12	0.05
**INDIRECT EFFECTS**					
Constraints from CW	0.08	0.02	4.35^**^	0.05	0.12
Constraints from IS	0.30	0.04	7.81^**^	0.23	0.38
TOTAL	0.38	0.04	8.52^**^	0.30	0.47

*N = 362*.

** *p < 0.01*.

*CI, Confidence interval; S.E., Standard error; CW, Coworkers; IS, The immediate supervisor. Control variable = Perceptions of overall constraints on the job in Time 1*.

## Discussion

Given the importance of situational strength in predicting employees' behavior and the assumption that situational strength can be exerted on employees by multiple sources simultaneously, it is important to assess how employees develop perceptions of overall situational strength on the job. The current paper sought to answer this question with a focus on situational strength from both distal (i.e., top management) and proximal (i.e., coworkers and the immediate supervisor) sources. We moreover examined the impact of these three sources with regard to two facets of situational strength: clarity and constraints. Confirmatory factor analyses showed that employees distinguished between the various sources of situational strength. Relative importance analyses showed that, as hypothesized, perceptions of both clarity and constraints from each of the three sources explained unique variance in the perceptions of overall situational strength.

With regard to the relative importance of situational strength from the sources, as hypothesized, employees attached more importance to constraints from proximal sources compared to constraints from the distal source. Contrary to expectations, however, employees attached more importance to clarity from the *distal* sources compared to clarity from proximal sources. Finally, as expected, proximal sources mediated the effect of the distal source on overall situational strength for both clarity and constraints.

How might the unsupportive relative importance results in the case of clarity be explained? In other words, why does clarity from the distal source have a greater impact than clarity from the proximal sources on overall clarity? A way to approach this question is to build on our previous discussions on the nature of situational strength emanating from the sources.

The impact of top management on the employee may operate through multiple channels. For example, as alluded to previously, top management may shape organizational arrangements such as formal reward systems, technological factors such as work flow processes, and even physical settings such as architectural design (Cardy and Selvarajan, 2001)—all of which influence employee behavior. Moreover, top management, charged with the strategic planning for the entire organization, creates a vision and establishes broad, long-term goals (Jacobs and McGee, 2001; Zaccaro and Klimoski, 2001). Top management's messages reflecting the organization's values and long-term objectives—perhaps communicated to every employee through periodic organization-wide emails—could help the employee see the “big picture” and put the employee's job tasks into context. In other words, whereas communications from the immediate supervisor may help the employee understand what to do, communications from top management may help the employee understand the overarching rationale for what he or she is doing. Consequently, the perceived clarity of informational cues emanating from top management may be more influential than the perceived clarity of informational cues from proximal sources in forming the employee's perceptions of overall clarity on the job.

These arguments are consistent with findings from job satisfaction research, which indicate that top management sometimes exerts an effect on the employee that is stronger than the effects of more proximal sources such as the immediate supervisor and coworkers (Dalal et al., 2011). The arguments are also consistent with findings from organizational communication studies that have examined the relative importance of employees' perceived communication relationships with and quality of information from coworkers, supervisors, and top management (Putti et al., 1990; Allen, 1992). These studies found that communication and information from top management had the greatest impact on employees' levels of organizational commitment (because of their impact on employees' sense of organizational membership and their perceptions of organizational climate).

Future research could therefore use qualitative content and discourse analysis techniques (e.g., Atay et al., 2015) to examine the themes that emerge from situational strength-related written and verbal messages received by employees from various organizational sources. Future research could also assess perceptions of situational strength from additional sources (e.g., non-social sources such as the nature of the work itself, Meyer et al., 2009; external social sources such as clients/customers, Oliver et al., 2016) as well as perceptions relevant to additional facets of situational strength not included in the current study (e.g., consistency and consequences, Meyer et al., 2014). Of course survey length constraints preclude the simultaneous examination of large numbers of *sources* of situational strength crossed with large numbers of *facets* of situational strength within any single study. Nonetheless, several avenues exist for future research. For instance, for a given facet of situational strength (e.g., clarity), future research could examine the relative importance of the multiple channels through which top management may attempt to influence employees' behavior (e.g., immediate supervisors vs. organizational pay/benefits systems vs. organizational promotion systems; Dalal et al., 2011).

Another avenue for future research is the role of individual differences on employees' perceptions of situational strength. In this regard, it is important to note that a person's perceptions of the strength of a situation are not solely a reflection of the objective characteristics of the situation associated with situational strength. Instead, perceptions of situational strength also reflect the characteristics of the perceiver. Specifically, objective characteristics of the situation are “filtered through [a person's] expectations, experiences, motives, and dispositions” (Meyer et al., 2014; p. 1,023). One aspect of employees' dispositions that may be of particular relevance here is personality strength, an individual difference construct to which we alluded in the Introduction, and which previous researchers have described as “the other side of the strong vs. weak situation coin” (Locke and Latham, 2004; p. 395). Based on this perspective, personality strength, indicated by an individual's tendency to behave in uniform ways across situations (no matter what the situation requires), would reduce the level of perceived situational strength in a given situation (Dalal et al., 2015). Other individual differences could influence employees' perceived psychological distance between themselves and the various sources of situational strength, and, in turn, could determine how employees combine situational strength emanating from these sources. For example, employees' levels of social dominance orientation (i.e., the tendency to endorse intergroup hierarchies; Pratto et al., 1994) could impact their perceptions of social distance between different hierarchical levels in the organization. Future research could therefore draw from the personality and social psychology literatures to examine the role of individual differences as antecedents of perceptions of situational strength.

## Limitations and conclusion

A putative limitation of this study is that the data were collected via self-report measures. However, same-source bias was ameliorated by collecting data at two time points (Podsakoff et al., 2003). Moreover, unlike some self-report data, situational strength data are unlikely to be influenced appreciably by socially desirable responding. Perhaps more importantly, the choice of self-report data was driven by the study's focus on nuanced employee perceptions: specifically, perceptions of situational strength emanating from multiple sources. For perceptual data such as these, self-reports are “not only justifiable but probably necessary” due to the limited insight that observers have into people's perceptions (Chan, 2009; p. 326). In particular, the validity of other-reports of perceptions rests on three dubious assumptions: (a) the focal person's perceptions translate well into observable behavior, (b) other people regularly have the opportunity to observe this perception-relevant behavior, and (c) observers are accurately able to back-translate a specific behavior into a specific valence relevant to a specific perception (Chan, 2009). Because none of these assumptions is likely to hold true, other-reports of people's perceptions cannot substitute for self-reports. It would, however, be interesting to examine the extent of (dis)agreement between self- and other-reports of situational strength from a source (e.g., immediate supervisor) as a variable of interest in and of itself. This, in turn, would lead to an emphasis on the factors that influence (dis)agreement. For instance, we suspect that the clarity of supervisor-to-subordinate communication is adjudged to be higher by the supervisor than by the subordinate. Moreover, we suspect that this disagreement is likely to be lower when both supervisor and subordinate score high rather than low on interpersonal skills such as perspective-taking, and when the supervisor and subordinate have had considerable experience working together. Future research should address questions such as these.

From a practical standpoint, the current research provides insight into the optimal location for situational-strength-related interventions. For instance, Human Resource Management interventions to decrease perceived constraints, and therefore increase the extent of dispositional discretion, would be more fruitfully targeted at proximal sources such as the immediate supervisor than at distal sources such as top management. In contrast, top management can have an outsized impact on employees' perceptions through the situational strength facet of clarity because communication about the organization's strategic plan and resultant policies may spur greater perceived clarity than the immediate supervisor's attempts to turn top management's policies into quotidian procedures to be followed by the employee. In a related vein, while organizations communicate the strategic plan and resultant policies through top management, they should also be mindful of the needs of key frontline employees (e.g., allies that we alluded to in the Introduction section) and immediate supervisors, who will be transmitting the effect of situational strength from top management. When necessary, these individuals should be trained on communicating new procedures and policies.

Finally, each social source of situational strength should consider the situational strength implications of its actions. For instance, several aspects of the organization's human resources management system (e.g., the electronic performance monitoring system, the telework policy) are likely to have large effects in terms of situational strength (Dalal and Meyer, 2012). Accordingly, top management should consider whether each new policy is aligned with the level of situational strength top management wishes organizational employees to experience.

## Ethics statement

We obtained human subjects approval from George Mason University's Institutional Review Board.

## Author contributions

RD developed the broad rationale for the paper and some of the research questions. BA, RD, AT, and SH fleshed out the theoretical foundation, improved, and added to the research questions, designed the study, and selected the instruments. All authors contributed to data collection. BA, ZS, AM, and SH contributed to data analysis. All authors contributed to the interpretation of the results. BA, RD, and ZS contributed to manuscript writing. AM, AT, and SH provided critical reviews for, and helped with the editing of, the manuscript prior to submission. BA, RD, ZS, AM, and SH contributed to manuscript revisions subsequent to reviewer feedback.

### Conflict of interest statement

The authors declare that the research was conducted in the absence of any commercial or financial relationships that could be construed as a potential conflict of interest. The reviewer AP and handling Editor declared their shared affiliation.

## Footnotes

^1^Agreeableness is characterized by trust, straightforwardness, altruism, compliance, modesty, and tender-mindedness; conscientiousness is characterized by competence, order, dutifulness, achievement striving, self-discipline, and deliberation; extraversion is characterized by warmth, gregariousness, assertiveness, activity, excitement-seeking, and positive emotions; neuroticism is characterized by anxiety, angry hostility, depression, self-consciousness, impulsiveness, and vulnerability; and openness to experience is characterized by fantasy, appreciation of aesthetics, receptiveness to inner feelings, willingness to try different activities and consider new ideas, and readiness to reflect on values (Costa and McCrae, 1992; see also Judge et al., 2013).

^2^A very short time interval is more likely to suffer from carryover effects, whereas a very long interval increases the chance that a change in status could occur; a 2-week interval is believed to be a good balance between the two (Marx et al., 2003). A 2-week interval is also commonly chosen in psychological survey designs involving two waves of data (e.g., Hideg and Ferris, 2017).

^3^Main analyses were also conducted on a data set that excluded the four cases with less than 50% missing data (*N* = 359). The substantive conclusions did not change when we restricted the analyses to only the subset of respondents with complete data. Accordingly, we report the results obtained from analyses with listwise deletion of missing data.

^4^Vandenberg and Lance (2000) note that a factor variance/covariance invariance test could be used to accompany the test of configural variance or metric invariance. Vandenberg and Lance (2000) also note, however, that support for an overall variance/covariance invariance test indicates that tests for specific aspects of measurement equivalence are neither needed nor warranted; moreover, the rejection of the variance/covariance invariance hypothesis is uninformative of the particular source of measurement inequivalence. Accordingly, to evaluate measurement equivalence of the focal variables we tested a series of increasingly restrictive hypotheses.

^5^As alluded to in the Participants and Procedure section, in the Time 1 survey, we included questions assessing: (1) age, (2) number of years worked with the current immediate supervisor, (3) number of years worked with the current top management, (4) number of years completed in the current organization, (5) number of years completed in the current job, (6) gender (male; female; other), (7) whether the respondent considers himself or herself to be a part of the organization's top management (yes; no), and (8) whether the respondent considers his or her immediate supervisor to be a part of the organization's top management (yes; no). After controlling for the continuous variables (i.e., variables 1–5), in addition to the original control variable of overall situational strength at Time 1, substantive conclusions from the RWA and mediation analyses were identical to those reported in the Results section. For the non-continuous variables (i.e., variables 6-8), we conducted the RWA and the mediation analyses separately for each group (using only male vs. female in the case of gender): If results did not vary across groups, then we can conclude that the control variables had no influence on the focal analyses. Results did not vary across the two groups of variables 6–8 for all but one analysis. The one exception is that the mediating effect of constraints from coworkers on the relationship between constraints from top management and overall constraints was stronger for the respondents who considered their immediate supervisors to be top management. This is in line with our overall findings (and field theory; Lewin, 1939, 1943, 1951): The influence from top management should be stronger if top management includes one's immediate supervisor. Besides, the mediating effect was established regardless of whether an employee considered his or her immediate supervisor to be top management. Readers who are interested in the version of the focal analyses that include the control variables can contact the first author.

## References

[B1] Adams J. S. (1976). The structure and dynamics of behavior in organizational boundary roles, in Handbook of Industrial and Organizational Psychology, ed Dunnette M. D. (Chicago, IL: Rand McNally), 321–355.

[B2] Aldrich H. Herker D. (1977). Boundary spanning roles and organization structure. Acad. Manage. Rev. 2, 217–230.

[B3] Allen M. W. (1992). Communication and organizational commitment: perceived organizational support as a mediating factor. Commun. Q. 40, 357–367. 10.1080/01463379209369852

[B4] Allen T. (1977). Managing the Flow of Technology. Cambridge, MA: MIT Press.

[B5] Amir O. Rand D. G. Gal Y. K. (2012). Economic games on the internet: the effect of $1 stakes. PLoS ONE 7:e31461. 10.1371/journal.pone.0031461 22363651 PMC3283743

[B6] Atay C. Conway E. R. Angus D. Wiles J. Baker R. Chenery H. J. (2015). An automated approach to examining conversational dynamics between people with dementia and their careers. PLoS ONE 10:e0144327 10.1371/journal.pone.0144327 26658135 PMC4675547

[B7] Barrick M. R. Mount M. K. (1993). Autonomy as a moderator of the relationships between the Big Five personality dimensions and job performance. J. Appl. Psychol. 78, 111–118. 10.1037/0021-9010.78.1.111

[B8] Becker T. E. (1992). Foci and bases of commitment: are they distinctions worth making? Acad. Manage. J. 35, 232–244. 10.2307/256481

[B9] Becker T. E. (2009). Interpersonal commitments, in Commitment in Organizations: Accumulated Wisdom and New Directions, eds Klein H. J. Becker T. E. Meyer J. P. (New York, NY: Routledge; Taylor and Francis Group), 137–178.

[B10] Becker T. E. Billings R. S. (1993). Profiles of commitment: an empirical test. J. Organ. Behav. 14, 177–190. 10.1002/job.4030140207

[B11] Becker T. E. Billings R. S. Eveleth D. M. Gilbert N. L. (1996). Foci and bases of employee commitment: implications for job performance. Acad. Manage. J. 39, 464–482. 10.2307/256788

[B12] Berson Y. Avolio B. J. (2004). Transformational leadership and the dissemination of organizational goals: a case study of a telecommunication firm. Leadersh. Q. 15, 625–646. 10.1016/j.leaqua.2004.07.003

[B13] Blau P. M. Scott W. R. (1962). Formal Organizations: A Comparative Approach. San Francisco, CA: Chandler.

[B14] Bloom M. (1999). The performance effects of pay dispersion on individuals and organizations. Acad. Manage. J. 42, 25–40. 10.2307/256872

[B15] Bowling N. A. Khazon S. Meyer R. D. Burrus C. J. (2015). Situational strength as a moderator of the relationship between job satisfaction and job performance: a meta-analytic examination. J. Bus. Psychol. 30, 89–104. 10.1007/s10869-013-9340-7

[B16] Buhrmester M. Kwang T. Gosling S. D. (2011). Amazon's Mechanical Turk: a new source of inexpensive, yet high-quality, data? Perspect. Psychol. Sci. 6, 3–5. 10.1177/1745691610393980 26162106

[B17] Cardy R. L. Selvarajan T. T. (2001). Management interventions, in Handbook of Industrial, Work and Organizational Psychology (Vol. 2: Organizational Psychology), eds Anderson N. Ones D. S. Sinangil H. K. Viswesvaran C. (London: Sage Publications), 346–376.

[B18] Casler K. Bickel L. Hackett E. (2013). Separate but equal? A comparison of participants and data gathered via Amazon's MTurk, social media, and face-to-face behavioral testing. Comput. Hum. Behav. 29, 2156–2160. 10.1016/j.chb.2013.05.009

[B19] Chan D. (2009). So why ask me? Are self-report data really that bad?, in Statistical and Methodological Myths and Urban Legends: Doctrine, Verity and Fable in the Organizational Sciences, eds Lance C. E. Vandenberg R. J. (New York, NY: Routledge), 309–336.

[B20] Chiaburu D. S. Harrison D. A. (2008). Do peers make the place? Conceptual synthesis and meta-analysis of coworker effects on perceptions, attitudes, OCBs, and performance. J. Appl. Psychol. 93, 1082–1103. 10.1037/0021-9010.93.5.1082 18808227

[B21] Cole D. A. Maxwell S. E. (2003). Testing mediational models with longitudinal data: questions and tips in the use of structural equation modeling. J. Abnorm. Psychol. 112, 558–577. 10.1037/0021-843X.112.4.558 14674869

[B22] Cooper W. H. Withey M. J. (2009). The strong situation hypothesis. Pers. Soc. Psychol. Rev. 13, 62–72. 10.1177/1088868308329378 19144905

[B23] Costa P. T. McCrae R. R. (1992). NEO PI-R Professional Manual. Odessa, FL: Psychological Assessment Resources.

[B24] Crump M. J. C. McDonnell J. V. Gureckis T. M. (2013). Evaluating Amazon's Mechanical Turk as a tool for experimental behavioral research. PLoS ONE 8:e57410. 10.1371/journal.pone.0057410 23516406 PMC3596391

[B25] Dalal R. S. (2005). A meta-analysis of the relationship between organizational citizenship behavior and counterproductive work behavior. J. Appl. Psychol. 90, 1241–1255. 10.1037/0021-9010.90.6.1241 16316277

[B26] Dalal R. S. Meyer R. D. (2012). The implications of situational strength for HRM, in The Encyclopedia of Human Resource Management, Vol. 3, eds Rothwell W. J. Benscoter G. M. (San Francisco, CA: Pfeiffer/Wiley), 298–306.

[B27] Dalal R. S. Bashshur M. R. Credé M. (2011). The forgotten facet: employee satisfaction with management above the level of immediate supervision. Appl. Psychol. 60, 183–209. 10.1111/j.1464-0597.2010.00431.x

[B28] Dalal R. S. Bhave D. P. Fiset J. (2014). Within-person variability in job performance: a theoretical review and research agenda. J. Manage. 40, 1396–1436. 10.1177/0149206314532691

[B29] Dalal R. S. Meyer R. D. Bradshaw R. P. Green J. P. Kelly E. D. Zhu M. (2015). Personality strength and situational influences on behavior: A conceptual review and research agenda. J. Manag. 41, 261–287. 10.1177/0149206314557524

[B30] Farrell A. D. (1994). Structural equation modeling with longitudinal data: strategies for examining group differences and reciprocal relationships. J. Consult. Clin. Psychol. 62, 477–487. 10.1037/0022-006X.62.3.477 8063974

[B31] Fast N. J. Sivanathan N. Mayer N. D. Galinsky A. D. (2012). Power and overconfident decision-making. Organ. Behav. Hum. Decis. Process. 117, 249–260. 10.1016/j.obhdp.2011.11.009

[B32] Gelfand M. J. Nishii L. H. Raver J. L. (2006). On the nature and importance of cultural tightness-looseness. J. Appl. Psychol. 91, 1225–1244. 10.1037/0021-9010.91.6.1225 17100480

[B33] Giacopelli N. M. Simpson K. M. Dalal R. S. Randolph K. L. Holland S. J. (2013). Maximizing as a predictor of job satisfaction and performance: a tale of three scales. Judgm. Decis. Mak. 8, 448–469.

[B34] Gouldner A. W. (1957). Cosmopolitans and locals: toward an analysis of latent social roles—I. Adm. Sci. Q. 2, 281–306. 10.2307/2391000

[B35] Gouldner A. W. (1958). Cosmopolitans and locals: toward an analysis of latent social roles—II. Adm. Sci. Q. 2, 444–480. 10.2307/2390795

[B36] Hackman J. R. Oldham G. R. (1974). The Job Diagnostic Survey: An Instrument for the Diagnosis of Jobs and the Evaluation of Job Redesign Projects (Tech. Rep. No. 4). New Haven, CT: Yale University Department of Administrative Sciences.

[B37] Halevy N. Chou E. Y. Galinsky A. D. (2011). A functional model of hierarchy: why, how, and when vertical differentiation enhances group performance. Organ. Psychol. Rev. 1, 32–52. 10.1177/2041386610380991

[B38] Halevy N. Chou E. Y. Galinsky A. D. Murnighan J. K. (2012). When hierarchy wins. Soc. Psychol. Personal. Sci. 3, 398–406. 10.1177/1948550611424225

[B39] Hayes A. F. (2013). Introduction to Mediation, Moderation, and Conditional Process Analysis. New York, NY: The Guilford Press.

[B40] Herzberg F. Mausner B. Peterson R. D. Capwell D. F. (1957). Job Attitudes: Review of Research and Opinion. Pittsburgh, PA: Psychological Service of Pittsburgh.

[B41] Hideg I. Ferris D. L. (2017). Dialectical thinking and fairness-based perspectives of affirmative action. J. Appl. Psychol. 102, 782–801. 10.1037/apl0000207 28150989

[B42] Holden C. J. Dennie T. Hicks A. D. (2013). Assessing the reliability of the M5-120 on Amazon's Mechanical Turk. Comput. Hum. Behav. 29, 1749–1754. 10.1016/j.chb.2013.02.020

[B43] Huang J. L. Curran P. G. Keeney J. Poposki E. M. DeShon R. P. (2012). Detecting and deterring insufficient effort responding to surveys. J. Bus. Psychol. 27, 99–114. 10.1007/s10869-011-9231-8

[B44] Ipeirotis P. G. (2010). Demographics of Mechanical Turk (March 2010). NYU Working Paper No. CEDER-10-01. Available online at: http://ssrn.com/abstract=1585030

[B45] Jackson P. R. Wall T. D. Martin R. Davids K. (1993). New measures of job control, cognitive demand, and production responsibility. J. Appl. Psychol. 78, 753–762. 10.1037/0021-9010.78.5.753

[B46] Jacobs T. O. McGee M. L. (2001). Competitive advantage: conceptual imperatives for executives, in The Nature of Organizational Leadership: Understanding the Performance Imperatives Confronting Today's Leaders eds Zaccaro S. J. Klimoski R. J. (San Francisco, CA: Jossey-Bass), 42–78.

[B47] Jaques E. (1976). A General Theory of Bureaucracy. New York, NY: Heinemann; Halsted Press.

[B48] Johns G. (2006). The essential impact of context on organizational behavior. Acad. Manage. Rev. 31, 386–408. 10.5465/AMR.2006.20208687

[B49] Johnson J. A. (2005). Ascertaining the validity of individual protocols from Web-based personality inventories. J. Res. Pers. 39, 103–129. 10.1016/j.jrp.2004.09.009

[B50] José I. J. Hermida R. Vega R. P. Chen T. R. Hale A. Dalal R. S. (2011). When preferred and actual levels of situational strength differ, in Extensions and Applications of Situational Strength in the Organizational Sciences. Symposium Conducted at the Meeting of the Academy of Management, ed Meyer R. D. (Chair), San Antonio, TX.

[B51] Judge T. A. Locke E. A. (1993). Effect of dysfunctional thought processes on subjective well-being and job satisfaction. J. Appl. Psychol. 78, 475–490. 10.1037/0021-9010.78.3.475

[B52] Judge T. A. Zapata C. P. (2015). The person-situation debate revisited: effect of situation strength and trait activation on the validity of the big five personality traits in predicting job performance. Acad. Manage. J. 58, 1149–1179. 10.5465/amj.2010.0837

[B53] Judge T. A. Rodell J. B. Klinger R. L. Simon L. S. Crawford E. R. (2013). Hierarchical representations of the five-factor model of personality in predicting job performance: integrating three organizing frameworks with two theoretical perspectives. J. Appl. Psychol. 98, 875–925. 10.1037/a0033901 24016206

[B54] Kanfer R. (1991). Motivation theory and industrial and organizational psychology, in Handbook of Industrial and Organizational Psychology, Vol. 1, eds Dunnette M. D. Hough L. M. (Palo Alto, CA: Consulting Psychologists Press), 75–170.

[B55] Katz D. Kahn R. L. (1978). The Social Psychology of Organizations, 2nd Edn. New York, NY: John Wiley and Sons.

[B56] Kline R. B. (2011). Principles and Practice of Structural Equation Modeling, 3rd Edn. New York, NY: Guilford Press.

[B57] Kluger A. N. DeNisi A. (1996). The effects of feedback interventions on performance: A historical review, a meta-analysis, and a preliminary feedback intervention theory. Psychol. Bull. 119, 254–284. 10.1037/0033-2909.119.2.254

[B58] LeBreton J. M. Hargis M. B. Griepentrog B. Oswald F. L. Ployhart R. E. (2007). A multidimensional approach for evaluating variables in organizational research and practice. Pers. Psychol. 60, 475–498. 10.1111/j.1744-6570.2007.00080.x

[B59] Lee S. Dalal R. S. (2016). Climate as situational strength: safety climate strength as a cross-level moderator of the relationship between conscientiousness and safety behaviour. Eur. J. Work Organ. Psychol. 25, 120–132. 10.1080/1359432X.2014.987231

[B60] Lewin K. (1939). Field theory and experiment in social psychology: concepts and methods. Am. J. Sociol. 44, 868–896. 10.1086/218177

[B61] Lewin K. (1943). Defining the “field at a given time.” Psychol. Rev. 50, 292–310. 10.1037/h0062738

[B62] Lewin K. (1951). Field Theory in Social Sciences. New York, NY: Harper & Row.

[B63] Locke E. A. Latham G. P. (2004). What should we do about motivation theory? six recommendations for the twenty-first century. Acad. Manage. Rev. 29:388 10.5465/amr.2004.13670974

[B64] Locke E. A. (1976). The nature and causes of job satisfaction, in Handbook of Industrial and Organizational Psychology, Vol. 1, ed Dunnette M. D. (Chicago, IL: Rand McNally), 1297–1343.

[B65] MacKinnon D. P. Fairchild A. J. Fritz M. S. (2007). Mediation analysis. Annu. Rev. Psychol. 58, 593–614. 10.1146/annurev.psych.58.110405.085542 16968208 PMC2819368

[B66] Marx R. G. Menezes A. Horovitz L. Jones E. C. Warren R. F. (2003). A comparison of two time intervals for test-retest reliability of health status instruments. J. Clin. Epidemiol. 56, 730–735. 10.1016/S0895-4356(03)00084-2 12954464

[B67] Mayer D. M. Kuenzi M. Greenbaum R. Bardes M. Salvador R. B. (2009). How low does ethical leadership flow? Test of a trickle-down model. Organ. Behav. Hum. Decis. Process. 108, 1–13. 10.1016/j.obhdp.2008.04.002

[B68] McCrae R. R. Costa P. T. (1985). Updating Norman's “adequacy taxonomy”: intelligence and personality dimensions in natural language and in questionnaires. J. Pers. Soc. Psychol. 49, 710–721. 10.1037/0022-3514.49.3.710 4045699

[B69] McGrath R. E. Mitchell M. Kim B. H. Hough L. (2010). Evidence for response bias as a source of error variance in applied assessment. Psychol. Bull. 136, 450–470. 10.1037/a0019216 20438146

[B70] Meyer R. D. Dalal R. S. Bonaccio S. (2009). A meta-analytic investigation into the moderating effects of situational strength on the conscientiousness-performance relationship. J. Organ. Behav. 30, 1077–1102. 10.1002/job.602

[B71] Meyer R. D. Dalal R. S. Hermida R. (2010). A review and synthesis of situational strength in the organizational sciences. J. Manage. 36, 121–140. 10.1177/0149206309349309

[B72] Meyer R. D. Dalal R. S. Jose I. J. Hermida R. Chen T. R. Vega R. P. (2014). Measuring job-related situational strength and assessing its interactive effects with personality on voluntary work behavior. J. Manage. 40, 1010–1041. 10.1177/0149206311425613

[B73] Mischel W. (1968). Consistency and specificity in behavior, in Personality and Assessment, ed Mischel W. (New York, NY: Wiley), 13–39.

[B74] Mischel W. (1977). The interaction of person and situation, in Personality at the Crossroads: Current Issues in Interactional Psychology, eds Magnusson D. Endler N. S. (Hillsdale, NJ: Lawrence Erlbaum), 333–352.

[B75] Murphy K. R. (2005). Why don't measures of broad dimensions of personality perform better as predictors of job performance? Hum. Perform. 18, 343–357. 10.1207/s15327043hup1804_2

[B76] Oliver T. Hausdorf P. Lievens F. Conlon P. (2016). Interpersonal dynamics in assessment center exercises: effects of role player portrayed disposition. J. Manage. 42, 1992–2017. 10.1177/0149206314525207

[B77] Osborn R. N. Hunt J. G. Jauch L. R. (2002). Toward a contextual theory of leadership. Leadersh. Q. 13, 797–837. 10.1016/S1048-9843(02)00154-6

[B78] Peters L. H. O'Connor E. J. (1980). Situational constraints and work outcomes: the influence of a frequently overlooked construct. Acad. Manage. Rev. 5, 391–397.

[B79] Podsakoff P. M. MacKenzie S. B. Bommer W. H. (1996). Meta-analysis of the relationships between Kerr and Jermier's substitutes for leadership and employee job attitudes, role perceptions, and performance. J. Appl. Psychol. 81, 380–399. 10.1037/0021-9010.81.4.380 8751455

[B80] Podsakoff P. M. MacKenzie S. B. Lee J. Y. Podsakoff N. P. (2003). Common method biases in behavioral research: a critical review of the literature and recommended remedies. J. Appl. Psychol. 88, 879–903. 10.1037/0021-9010.88.5.879 14516251

[B81] Pratto F. Sidanius J. Stallworth L. M. Malle B. F. (1994). Social dominance orientation: a personality variable predicting social and political attitudes. J. Pers. Soc. Psychol. 67, 741–763. 10.1037/0022-3514.67.4.741

[B82] Putti J. M. Aryee S. Phua J. (1990). Communication relationship satisfaction and organizational commitment. Group Organ. Stud. 15, 44–52. 10.1177/105960119001500104

[B83] Reichers A. E. (1985). A review and reconceptualization of organizational commitment. Acad. Manage. Rev. 10, 465–476. 10300307

[B84] Rizzo J. R. House R. J. Lirtzman S. I. (1970). Role conflict and ambiguity in complex organizations. Adm. Sci. Q. 15, 150 10.2307/2391486

[B85] Sabat I. E. Lindsey A. P. Membere A. Anderson A. Ahmad A. King E. (2014). Invisible disabilities: unique strategies for workplace allies. Ind. Organ. Psychol. 7, 259–265. 10.1111/iops.12145

[B86] Salancik G. R. Pfeffer J. (1978). A social information processing approach to job attitudes and task design. Adm. Sci. Q. 23, 224–253. 10.2307/2392563 10307892

[B87] Sias P. M. Cahill D. J. (1998). From coworkers to friends: the development of peer friendships in the workplace. Western J. Commun. 62, 273–299. 10.1080/10570319809374611

[B88] Smith P. C. Kendall L. Hulin C. L. (1969). The Measurement of Satisfaction in Work and Retirement. Chicago, IL: Rand McNally.

[B89] Snyder M. Ickes W. (1985). Personality and social behavior, in Handbook of Social Psychology, 3rd Edn, eds Lindzey G. Aronson E. (New York, NY: Random House), 883–948.

[B90] Tonidandel S. LeBreton J. M. (2015). RWA Web: a free, comprehensive, web-based, and user-friendly tool for relative weight analyses. J. Bus. Psychol. 30, 207–216. 10.1007/s10869-014-9351-z

[B91] Tonidandel S. LeBreton J. M. Johnson J. W. (2009). Determining the statistical significance of relative weights. Psychol. Methods 14, 387–399. 10.1037/a0017735 19968399

[B92] Vandenberg R. J. Lance C. E. (2000). A review and synthesis of the measurement invariance literature: suggestions, practices, and recommendations for organizational research. Organ. Res. Methods 3, 4–70. 10.1177/109442810031002

[B93] Zaccaro S. J. Klimoski R. (2001). The nature of organizational leadership: an introduction, in The Nature of Organizational Leadership: Understanding the Performance Imperatives Confronting Today's Leaders, eds Zaccaro S. J. Klimoski R. J. (San Francisco, CA: Jossey-Bass), 3–41.

